# Efficacy and safety of 2 Hz versus 100 Hz electroacupuncture for pain-dominant shoulder periarthritis: a study protocol for a single-center randomized controlled trial

**DOI:** 10.3389/fmed.2026.1924469

**Published:** 2026-08-27

**Authors:** Chengyu Liu, Shiwei Zhuang, Yi Shang, Xiangzhen Miao, Hongfeng Wang, Bing Yan

**Affiliations:** 1 School of Rehabilitation Medicine (School of Integrated Chinese and Western Medicine), Changchun University of Chinese Medicine, Changchun, China; 2 The Third Clinical Hospital Affiliated to Changchun University of Chinese Medicine, Changchun, China; 3 School of Acupuncture-Moxibustion and Tuina, Changchun University of Chinese Medicine, Changchun, China; 4 School of Chinese Materia Medica, Beijing University of Chinese Medicine, Beijing, China; 5 Northeast Asia Institute of Traditional Chinese Medicine, Changchun University of Chinese Medicine, Changchun, China; 6 Institute of Basic Research in Clinical Medicine, China Academy of Chinese Medical Sciences, Beijing, China

**Keywords:** electroacupuncture, frequency, randomized controlled trial, shoulder periarthritis, study protocol

## Abstract

**Background:**

Pain-dominant shoulder periarthritis is characterized by shoulder pain, restricted range of motion (ROM), and impaired daily function. Electroacupuncture (EA) is a controllable acupuncture modality used for shoulder pain disorders; however, direct evidence comparing 2 Hz and 100 Hz EA in this disease stage remains limited.

**Objective:**

This study aims to compare the efficacy and safety of low-frequency EA (2 Hz) and high-frequency EA (100 Hz) for pain-dominant shoulder periarthritis.

**Methods:**

This is a single-center, participant- and outcome assessor-blinded, parallel-group randomized controlled trial (RCT) to be conducted at the Third Clinical Hospital Affiliated to Changchun University of Chinese Medicine. A total of 152 participants who meet the diagnostic criteria for pain-dominant shoulder periarthritis and the traditional Chinese medicine (TCM) meridian differentiation criteria for the Hand Shaoyang meridian pattern will be enrolled. Participants will be randomly assigned in a 1:1 ratio to the 2 Hz EA group or the 100 Hz EA group. Both groups will receive the same acupuncture prescription, treatment procedures, and co-interventions, with EA frequency as the only planned between-group difference. Treatment will be administered once daily for 5 consecutive days per course, with a 2-day interval between courses, for a total of 10 sessions. Outcomes will be assessed at baseline and at 2, 8, and 24 weeks after treatment initiation. The primary outcome will be overall shoulder pain intensity measured using a 10-cm visual analog scale (VAS) at T1. The primary comparison will be the adjusted mean difference in the T1 overall VAS score between the two groups, with baseline overall VAS included as a covariate. Secondary outcomes will include component-specific VAS ratings for resting pain, activity-related pain, and nocturnal pain, the Shoulder Pain and Disability Index (SPADI), the Constant-Murley Score (CMS), and shoulder ROM. Adverse events (AEs) and serious adverse events (SAEs) will be recorded throughout the study.

**Discussion:**

This trial may provide clinical evidence to inform EA frequency selection and parameter standardization for pain-dominant shoulder periarthritis.

**Clinical trial registration:**

International Traditional Medicine Clinical Trial Registry, https://itmctr.ccebtcm.org.cn, identifier ITMCTR2026000862.

## 1 Introduction

Shoulder periarthritis, also known as frozen shoulder or adhesive capsulitis, is a common shoulder disorder characterized by shoulder pain, restricted active range of motion (AROM) and passive range of motion (PROM), and shoulder-related functional impairment (1). The main pathological processes include capsular inflammation, fibrosis, adhesion, and contracture, which can reduce joint cavity volume and limit shoulder mobility (2).

The disease usually follows a staged clinical course and is commonly described as progressing through a pain-dominant stage, a stiffness stage, and a recovery stage. The pain-dominant stage is mainly characterized by resting pain, nocturnal pain, and activity-related pain, and may also involve a rapid decline in shoulder ROM. These symptoms can substantially affect sleep and daily activities, such as dressing, combing hair, reaching overhead, and shoulder abduction (3). Therefore, treatment at this stage should focus on prompt pain relief, preservation of tolerable shoulder movement, and prevention of persistent functional limitation.

Current conservative treatments for shoulder periarthritis include oral analgesic and anti-inflammatory medications, physical therapy, range-of-motion exercises, and rehabilitation interventions (4). For patients with prominent pain or more severe functional limitation, local corticosteroid injection, hydrodilatation, or other interventional treatments may also be considered (5). These approaches can relieve pain and improve shoulder mobility in the early stage. However, uncertainty remains regarding the optimal timing, treatment combinations, patient responses, and individual suitability of different interventions. Pharmacological and injection-based treatments may also be limited by injection-related discomfort, potential adverse effects, and patient acceptability (6). Therefore, a safe, reproducible, and standardized non-pharmacological treatment strategy is still needed.

Acupuncture is widely used as a non-pharmacological therapy for pain conditions (7). For shoulder periarthritis and related shoulder pain disorders, acupuncture may reduce pain and improve function through multiple mechanisms, including modulation of pain signaling, neurotransmitter activity, inflammatory responses, and peripheral or central sensitization (8). Electroacupuncture combines traditional acupuncture with electrical stimulation delivered through needles inserted at selected acupoints. Compared with manual acupuncture, EA allows better standardization of stimulation frequency, waveform, intensity, and treatment duration (9). This feature makes EA suitable for evaluating the relationship between stimulation parameters and clinical effects.

Previous studies have suggested that EA may relieve shoulder periarthritis-related pain compared with manual acupuncture or sham controls (10, 11). Randomized trials have also indicated that EA may reduce pain and improve shoulder function or mobility in patients with frozen shoulder (11, 12). Its overall safety profile appears acceptable, with few serious AEs reported (13). With the accumulation of evidence on acupuncture for shoulder pain (14), relevant evidence-based guidelines have recommended acupuncture for reducing pain intensity and improving shoulder ROM at appropriate clinical stages (15).

Among EA parameters, stimulation frequency is a key and quantifiable variable. Different frequencies may produce analgesic effects through distinct neurochemical and pain-modulatory mechanisms (16). Low-frequency EA is generally considered to be associated with the release of endogenous opioid peptides involving *μ*- and *δ*-opioid receptor pathways, whereas high-frequency EA may involve *κ*-opioid receptor pathways and spinal pain modulation (17). On this basis, 2 Hz and 100 Hz are commonly used as representative low- and high-frequency stimulation patterns, respectively (18–20).

However, direct evidence from RCTs comparing 2 Hz and 100 Hz EA remains lacking in shoulder periarthritis, especially in the pain-dominant stage. Existing studies have mainly compared EA with manual acupuncture, usual care, or sham controls. The specific question of which frequency is more suitable for pain-dominant shoulder periarthritis has not been adequately addressed.

Accordingly, this study was designed as a single-center, participant- and outcome assessor-blinded, parallel-group RCT. It aims to compare the efficacy and safety of 2 Hz and 100 Hz EA for pain-dominant shoulder periarthritis. Pain intensity will be assessed as the primary outcome. Shoulder pain and disability, overall shoulder function, and shoulder ROM will be evaluated as secondary outcomes. This trial is expected to clarify potential differences between low- and high-frequency EA in early pain relief, functional improvement, and sustained treatment effects during follow-up. The findings may provide evidence to inform EA frequency selection and parameter standardization for pain-dominant shoulder periarthritis.

## 2 Methods and analysis

### 2.1 Study design

This is a single-center, participant- and outcome assessor-blinded, parallel-group RCT. The trial aims to compare the analgesic effects, functional benefits, and safety of low-frequency EA (2 Hz) and high-frequency EA (100 Hz) in patients with pain-dominant shoulder periarthritis. Participant recruitment and treatment will be conducted at the Third Clinical Hospital Affiliated to Changchun University of Chinese Medicine from March 2026 to October 2026. Follow-up and data collection will continue until the last participant completes the T3 assessment. A total of 152 eligible participants will be recruited and randomly assigned in a 1:1 ratio to the low-frequency EA group (2 Hz; *n* = 76) or the high-frequency EA group (100 Hz; *n* = 76).

**Figure 1:**
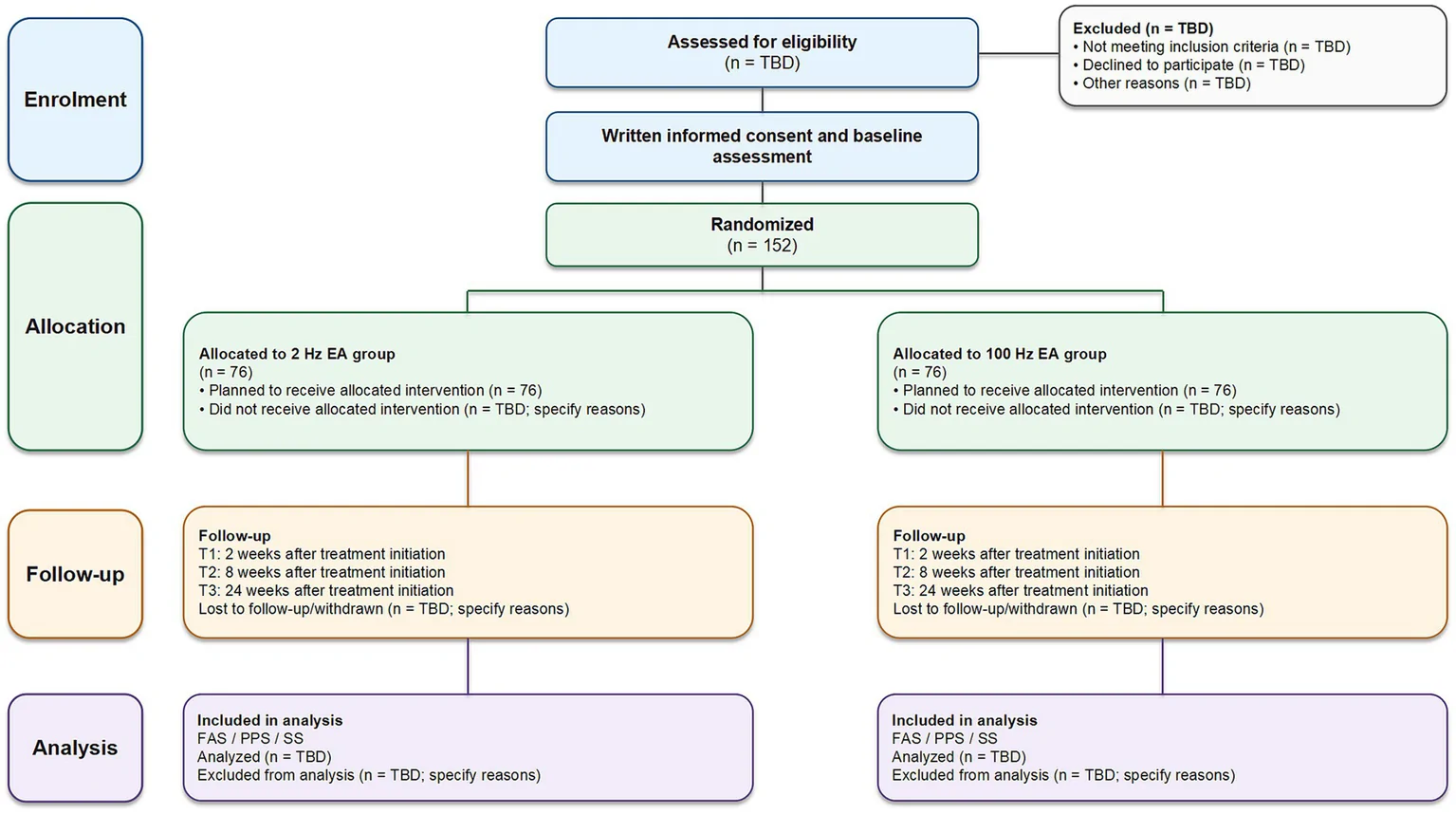
Study flow diagram. Values marked as “TBD” will be recorded during the trial and reported after trial completion. EA, electroacupuncture; FAS, full analysis set; PPS, per-protocol set; SS, safety set.

Four assessment time points will be used in this trial: T0 (baseline/before randomization), T1 (end of treatment, 2 weeks after treatment initiation), T2 (short-term follow-up, 8 weeks after treatment initiation), and T3 (medium-term follow-up, 24 weeks after treatment initiation). Outcomes related to pain, shoulder function, and shoulder ROM will be collected at each assessment time point. Adverse events and SAEs will be recorded throughout the trial. The primary outcome will be overall shoulder pain intensity measured using a 10-cm VAS at T1. The primary comparison will be the adjusted mean difference in the T1 overall VAS score between the two groups, with baseline overall VAS included as a covariate. Secondary outcomes will include component-specific VAS ratings for resting pain, activity-related pain, and nocturnal pain, the SPADI, the CMS, and shoulder ROM. ROM will include AROM and PROM. Table 1 summarizes the schedule of enrollment, interventions, and assessments. Figure 1 shows the study flowchart.

**Table 1:** Schedule of enrolment, interventions, and assessments.

Study phase	Screening period	T0	Treatment period	T1	T2	T3
Participant recruitment	×					
Pre-screening	×					
Informed consent	×					
Eligibility screening	×					
Western medical diagnostic criteria assessment	×					
TCM meridian differentiation assessment	×					
Inclusion/exclusion criteria review	×					
Medical history and physical examination	×					
Standard shoulder radiography and mandatory diagnostic ultrasonography	×					
Re-verification of pain-dominant status, including resting and nocturnal pain	×	×				
Baseline demographic data		×				
Baseline disease-related data		×				
Randomization		×				
Low-frequency EA group (2 Hz)			×			
High-frequency EA group (100 Hz)			×			
TDP irradiation			×			
VAS		×		×	×	×
Component-specific VAS ratings: resting, activity-related, and nocturnal pain		×		×	×	×
SPADI		×		×	×	×
CMS		×		×	×	×
Shoulder ROM (AROM/PROM)		×		×	×	×
Acupuncture sensation scale/sensation record			After each session			
EA device output level and within-session adjustment records			After each session			
Blinding success assessment				×		
Concomitant treatment records		×	Continuous	×	×	×
Rescue analgesic use records		×	Continuous	×	×	×
AE/SAE records		×	Continuous	×	×	×
Adherence and protocol deviation records			Continuous	×	×	×

× indicates that the item will be performed or assessed at the corresponding time point. Treatment period refers to weeks 1–2 after treatment initiation. T1, T2, and T3 refer to the end of treatment, 8 weeks after treatment initiation, and 24 weeks after treatment initiation, respectively. “After each session” indicates that the acupuncture sensation scale or sensation record will be completed after each treatment session. “Continuous” indicates ongoing recording from randomization to the final follow-up when applicable. Treatment consists of 10 sessions: once daily, 5 consecutive sessions per course, with a 2-day interval between courses. AE, adverse event; AROM, active range of motion; CMS, Constant–Murley Score; EA, electroacupuncture; PROM, passive range of motion; ROM, range of motion; SAE, serious adverse event; SPADI, Shoulder Pain and Disability Index; T0, baseline/before randomization; T1, end of treatment/2 weeks after treatment initiation; T2, 8 weeks after treatment initiation; T3, 24 weeks after treatment initiation; TCM, traditional Chinese medicine; TDP, specific electromagnetic spectrum lamp; VAS, Visual Analog Scale.

This study protocol follows the Standard Protocol Items: Recommendations for Interventional Trials (SPIRIT) 2025 reporting guideline (21), and the completed SPIRIT 2025 checklist is provided as Supplementary Material 1. Details of the acupuncture intervention will be reported with reference to Standards for Reporting Interventions in Clinical Trials of Acupuncture (STRICTA) (22). The trial will be conducted in accordance with the Declaration of Helsinki and relevant ethical requirements for clinical research. All candidate participants will be fully informed of the study purpose, trial procedures, potential benefits and risks, data confidentiality measures, and their right to withdraw before participation. Written informed consent will be obtained voluntarily from each participant. The study protocol has been approved by the Medical Ethics Committee of the Third Clinical Hospital Affiliated to Changchun University of Chinese Medicine (approval No. CZDSFYLL2025-030-01). The trial has been registered with the International Traditional Medicine Clinical Trial Registry (registration No. ITMCTR2026000862; https://itmctr.ccebtcm.org.cn).

### 2.2 Diagnostic criteria

The diagnostic criteria in this trial include Western medical diagnostic criteria for shoulder periarthritis and TCM meridian differentiation criteria. All participants must meet the Western medical diagnostic criteria for pain-dominant shoulder periarthritis and the meridian differentiation criteria for the Hand Shaoyang meridian pattern.

#### 2.2.1 Western medical diagnostic criteria

The Western medical diagnostic criteria for shoulder periarthritis are based on classic descriptions of frozen shoulder (23), the consensus definition of frozen shoulder (1), and relevant clinical practice guidelines (3). Participants who meet the following criteria will be diagnosed with pain-dominant shoulder periarthritis:

- Pain is present in the affected shoulder. The main manifestations include resting pain, nocturnal pain, or activity-related pain. The pain may gradually worsen or recur over time;
- Both active and PROM are limited in the affected shoulder. The limitation involves at least two movement directions. Commonly affected directions include external rotation, abduction, forward flexion, and extension;
- Pain and restricted movement lead to reduced shoulder function. Daily activities may be affected, including dressing, combing hair, reaching overhead, shoulder abduction, shoulder extension, or sleep;
- Pain, rather than stiffness, is the predominant clinical manifestation and is accompanied by reduced shoulder ROM. At formal screening and again at the baseline visit before randomization, the participant must report active resting pain or nocturnal pain during the preceding 24 h, with at least one of these pain components rated ≥3 cm on a 10-cm VAS. Patients whose current clinical presentation is dominated by stiffness and mechanical restriction without active resting or nocturnal pain will not be considered to have pain-dominant shoulder periarthritis;
- Standard shoulder radiography shows no fracture, dislocation, marked osteoarthritis, or other osseous structural lesion that could explain the current symptoms;
- Mandatory diagnostic ultrasonography of the affected shoulder shows no partial-thickness or full-thickness rotator cuff tear. Subacromial impingement syndrome, calcific tendinitis, cervicogenic shoulder pain, radiculopathy, tumor, infection, and other definite causes of shoulder pain are excluded on the basis of the medical history, physical examination, shoulder radiography, and diagnostic ultrasonography. Shoulder magnetic resonance imaging will be performed if the ultrasonographic findings are inconclusive or if additional structural pathology is suspected.

#### 2.2.2 TCM meridian differentiation criteria

The TCM meridian differentiation criteria are based on the classification and treatment of Loujianfeng in Acupuncture Therapeutics, a national planning textbook for higher education in TCM during the Fourteenth Five-Year Plan period (24). This trial will include participants with the Hand Shaoyang meridian pattern. Participants who meet the following criteria will be identified as having the Hand Shaoyang meridian pattern:

- Pain is mainly located on the lateral or posterolateral side of the shoulder and may radiate along the lateral side of the upper arm;
- Obvious tenderness is present near Jianliao (SJ14) or along the pathway of the Hand Shaoyang meridian;
- Pain is aggravated during shoulder abduction, elevation, or external rotation, with corresponding movement limitation;
- Local soreness, distension, tightness, discomfort, or aggravation after exertion may be present;
- The Hand Shaoyang meridian pattern is determined by the study physician after a comprehensive assessment of medical history, pain location, tender point distribution, and characteristics of shoulder movement limitation.

### 2.3 Inclusion criteria

Participants must meet all of the following inclusion criteria and none of the exclusion criteria listed in Section 2.4:

- They meet both the Western medical diagnostic criteria for pain-dominant shoulder periarthritis and the TCM meridian differentiation criteria for the Hand Shaoyang meridian pattern, as described in Section 2.2;
- They are 30–80 years of age, inclusive, regardless of sex;
- They have unilateral shoulder pain and restricted shoulder movement, with clear tender points around the affected shoulder;
- The disease duration is ≥4 weeks and <36 weeks. Because the timing of clinical stage transition varies among patients, eligibility will be determined according to the current clinical presentation rather than disease duration alone. Pain must remain the predominant clinical manifestation, and active resting pain or nocturnal pain must be present, with at least one of these components rated ≥3 cm on a 10-cm VAS at both formal screening and the baseline visit before randomization;
- The pain score on a 10-cm VAS is >3 at screening;
- Standard shoulder radiography shows no fracture, dislocation, marked osteoarthritis, or other osseous lesion that could explain the current symptoms, and mandatory diagnostic ultrasonography of the affected shoulder shows no partial-thickness or full-thickness rotator cuff tear or other clinically relevant soft-tissue lesion that could account for the presenting symptoms;
- They voluntarily agree to participate in the study and sign written informed consent, and are able to complete the treatment, outcome assessments, and follow-up procedures.

### 2.4 Exclusion criteria

Participants who meet the preliminary screening criteria will be excluded if any of the following conditions are present:

- Structural lesions are present in the affected shoulder, including fracture, dislocation, any partial-thickness or full-thickness rotator cuff tear, marked shoulder osteoarthritis, or a history of major surgery on the affected shoulder that could explain the current symptoms;
- Shoulder pain or shoulder dysfunction is caused by other definite etiologies, including subacromial impingement syndrome, calcific tendinitis, cervicogenic shoulder pain, radiculopathy, bone tumor, shoulder tuberculosis, infectious arthritis, or inflammatory arthropathy;
- Bilateral shoulder pain or marked bilateral restriction of shoulder movement is present, and the main affected side cannot be clearly determined. Participants will also be excluded if bilateral symptoms may affect outcome assessment;
- They have participated in another clinical trial within the previous month, or have received treatments within the previous 3 months that may substantially affect the evaluation of pain or shoulder function. These treatments include intra-articular shoulder injection, subacromial bursa injection, tendon sheath injection, nerve block, hydrodilatation, acupotomy, miniscalpel-needle therapy, or other interventional treatments;
- They have received acupuncture, EA, tuina, physical therapy, intensive rehabilitation training, or other treatments that may substantially affect the evaluation of shoulder pain or shoulder ROM within 2 weeks before screening;
- They have contraindications to acupuncture or EA, including rash, ulceration, or infection at the needling site; coagulation disorders or a high risk of bleeding; current anticoagulant use judged by the study physician to be unsuitable for acupuncture; a cardiac pacemaker or other implanted electronic device; or severe metal allergy to acupuncture needles;
- They have severe diseases of the heart, brain, liver, kidney, hematopoietic system, or other major systems. They will also be excluded if they have an active infectious disease, severe skin disease, or any condition judged by clinical assessment to make them unsuitable for the study intervention;
- They are pregnant, lactating, or planning pregnancy during the study period;
- They have a severe mental disorder, marked cognitive impairment, or language communication disorder that prevents them from understanding the study procedures or completing outcome assessments;
- They are expected to be unable to complete the treatment or follow-up assessments because of personal plans, relocation, work arrangements, or other practical reasons.

### 2.5 Recruitment process

Participants will be mainly recruited from the outpatient acupuncture clinic of the Third Clinical Hospital Affiliated to Changchun University of Chinese Medicine. The research team will consecutively screen potentially eligible patients attending the outpatient clinic. Recruitment information will also be released through hospital posters, the official hospital website, and the hospital WeChat public account. All recruitment materials will be approved by the ethics committee before use. These materials will include the study purpose, main eligibility criteria, trial procedures, potential benefits and risks, contact information, and participants’ rights.

Individuals interested in participating will first undergo pre-screening. Pre-screening may be conducted face to face, by telephone, or online. It will mainly assess age, main symptoms, disease duration, previous treatment history, and obvious exclusion factors. Individuals who are potentially eligible after pre-screening will be scheduled for a formal screening visit at the research clinic. Formal screening will be performed by trained research staff and study physicians and will include medical history taking, a standardized physical examination, preliminary assessment of shoulder ROM, standard shoulder radiography, and mandatory diagnostic ultrasonography of the affected shoulder. Ultrasonography will be performed by trained imaging personnel and will assess the integrity of the supraspinatus, infraspinatus, subscapularis, and teres minor tendons, as well as other relevant periarticular soft-tissue structures. Shoulder MRI will be obtained if the ultrasonographic findings are inconclusive or if another structural lesion is suspected. All imaging findings will be reviewed before eligibility is confirmed and randomization is performed. Final eligibility will be determined according to the diagnostic criteria in Section 2.2, the inclusion criteria in Section 2.3, and the exclusion criteria in Section 2.4. A screening log will be maintained. Screening results and reasons for non-enrollment will be recorded for all screened individuals.

Before any study-related procedure is performed, research staff will provide full information to each candidate participant. This information will include the study purpose, trial procedures, potential benefits and risks, alternative treatment options, data confidentiality measures, the right to withdraw, and protection of medical rights after withdrawal. Candidate participants will be given sufficient time to ask questions and consider participation. Individuals who voluntarily agree to participate will sign written informed consent and receive a unique study identification number. Participants who have signed informed consent and are confirmed as eligible will complete the baseline assessment before randomization. At the baseline visit and before randomization, the study physician will re-evaluate the participant’s current clinical stage. Pain-dominant status will be reconfirmed on the basis of the predominant clinical complaint and the presence and intensity of resting and nocturnal pain. Candidates whose symptoms have transitioned to a stiffness-dominant presentation or who no longer meet the pain-dominant criteria will not be randomized, and the reason for non-enrollment will be recorded in the screening log. Personal information will be used only for the purposes of this study and will be managed in a de-identified manner. Participants may withdraw from the study at any time without penalty. Reasons for withdrawal will be recorded in the screening, enrollment, and follow-up records when available.

### 2.6 Sample size calculation

The sample size was calculated using PASS version 2023. Although the primary analysis will use an analysis of covariance model with adjustment for baseline VAS, the sample size was conservatively estimated using a two-sided test for comparing the means of two independent groups, without assuming any efficiency gain from baseline adjustment.

A published randomized controlled trial involving patients with frozen shoulder reported a post-treatment standard deviation of approximately 1.9 cm for the 10-cm VAS in both active treatment groups (12). On this basis, the common standard deviation was conservatively set at 2.0 cm. Considering that the present trial compares two active EA frequency regimens, the smallest between-group difference of interest was set at 1.0 cm, corresponding to a standardized effect size of 0.50. This target between-group mean difference is distinct from the individual-level minimal clinically important difference, which reflects the magnitude of change perceived as beneficial by an individual participant. With a two-sided significance level of 0.05, statistical power of 0.80, and a 1:1 allocation ratio, 64 participants with evaluable primary-outcome data will be required in each group. Allowing for an anticipated attrition rate of 15%, 76 participants will be recruited per group. Therefore, the total planned sample size will be 152 participants.

### 2.7 Randomization and allocation concealment

Participants who meet the inclusion criteria, meet none of the exclusion criteria, and provide written informed consent will be randomized after the baseline assessment. They will be assigned in a 1:1 ratio to the low-frequency EA group (2 Hz) or the high-frequency EA group (100 Hz). The randomization sequence will be generated by an independent statistician using a computer-generated method. The statistician will not participate in participant recruitment, treatment, outcome assessment, or data analysis. Block randomization will be used. The block size will be determined and kept confidential by the independent statistician.

Allocation concealment will be implemented through a central randomization system. Randomization will be performed only after eligibility confirmation, informed consent, and baseline assessment have been completed. The allocation result will be available only to the treating acupuncturist responsible for delivering the intervention. The treating acupuncturist will not participate in outcome assessment or data analysis. Investigators, outcome assessors, data managers, and statistical analysts will have no access to the randomization sequence or the complete allocation list before database locking. The randomization sequence, allocation records, and system operation logs will be stored by independent personnel.

### 2.8 Blinding

This trial will use a participant- and outcome assessor-blinded design. Participants will be informed that the trial compares two EA frequency regimens that have been used in clinical practice. They will not be informed of their group allocation. The two groups will be kept consistent in acupoint selection, needles, treatment position, needle retention time, treatment setting, treatment procedures, and communication scripts. These measures will be used to minimize expectation bias.

EA produces perceptible sensations, including soreness, numbness, distension, heaviness, tingling, and slight local muscle twitching. Because different stimulation frequencies may produce different temporal sensations, complete participant blinding cannot be guaranteed. Participants will not be informed of the expected sensory characteristics of either frequency. The EA device display and control panel will be positioned outside the participant’s line of sight, and treating acupuncturists will avoid mentioning the selected frequency or describing frequency-specific sensations during treatment.

Treating acupuncturists cannot be blinded because they need to set the EA frequency. Therefore, they will not participate in participant screening, outcome assessment, data entry, data management, or statistical analysis.

Outcome assessors, data managers, and statistical analysts will remain blinded throughout the trial. During data entry and statistical analysis, the groups will be coded as group A and group B. The specific EA frequency corresponding to each code will not be revealed before database locking. All research personnel will receive standardized training before the trial begins and will follow standard operating procedures (SOPs) for blinded communication, treatment arrangements, outcome assessment, and data management. Before participant recruitment begins, a formal role-assignment and delegation log will be established to define the responsibilities of personnel involved in participant screening, eligibility assessment, intervention delivery, outcome assessment, data entry, data management, and statistical analysis. These roles will be assigned to different personnel wherever blinding requires separation. Treating acupuncturists will have access only to participant information and treatment parameters necessary for intervention delivery and will not have access to outcome-assessment data or the blinded analysis dataset. Treatment delivery and outcome assessment will be scheduled and conducted separately, and standardized communication scripts will be used in both treatment groups. Any event that may lead to unblinding will be recorded promptly. The record will include the time, reason, personnel involved, and management measures.

The success of participant blinding will be assessed at T1, at the end of treatment 2 weeks after treatment initiation. Participants will be asked whether they believe they received the 2 Hz regimen, the 100 Hz regimen, or are uncertain about their allocation. The reason for their judgment and their level of confidence on a 0–10 scale will also be recorded. Participants’ guesses will be cross-tabulated with their actual treatment allocation, and the proportions of correct, incorrect, and uncertain responses will be reported. If necessary, outcome assessors may also be surveyed to evaluate the success of assessor blinding. The participant blinding results, including the reasons for participants’ judgments and their confidence ratings, will be considered when interpreting the trial findings.

Safety will be monitored continuously throughout the trial. Emergency individual unblinding may be performed if a serious adverse event, marked disease aggravation, or another condition requiring urgent medical management based on group allocation occurs. Emergency unblinding must be requested by the investigator with a clear explanation of medical necessity and confirmed by the principal investigator or the trial steering committee. The participant’s actual group allocation will then be obtained through the central randomization system. Emergency unblinding will be limited to the relevant participant and will not affect the blinding of other participants or research personnel. All emergency unblinding events will be documented. The record will include the time of unblinding, reason, decision-making process, personnel involved, and subsequent management. The case report form and adverse event records will be updated accordingly.

### 2.9 Intervention

The acupuncture procedures in this trial will be performed by three licensed acupuncturists who have received standardized training. Quality control will be supervised throughout the trial by an associate chief physician with more than 20 years of clinical experience in acupuncture. The research team has developed and rehearsed a SOP and an operation checklist. The checklist covers acupoint location, needle selection, insertion angle and depth, needling manipulation, assessment of de qi, EA parameter setting, needle retention time, Teding Diancibo Pu (TDP) lamp irradiation, safety monitoring, and adverse event reporting. Except for the EA stimulation frequency, all intervention components, treatment settings, operating procedures, and communication scripts will be kept consistent between the two groups to ensure intervention consistency and reproducibility.

Participants will be placed in a lateral decubitus position with the affected side upward. A semi-recumbent position may be used when necessary. This position will allow adequate exposure of the affected shoulder and relevant upper-limb acupoint areas. The local skin will be routinely disinfected before needling. All treatments will be performed in a quiet treatment room with an appropriate ambient temperature. Unnecessary external stimulation will be avoided during treatment.

**Figure 2:**
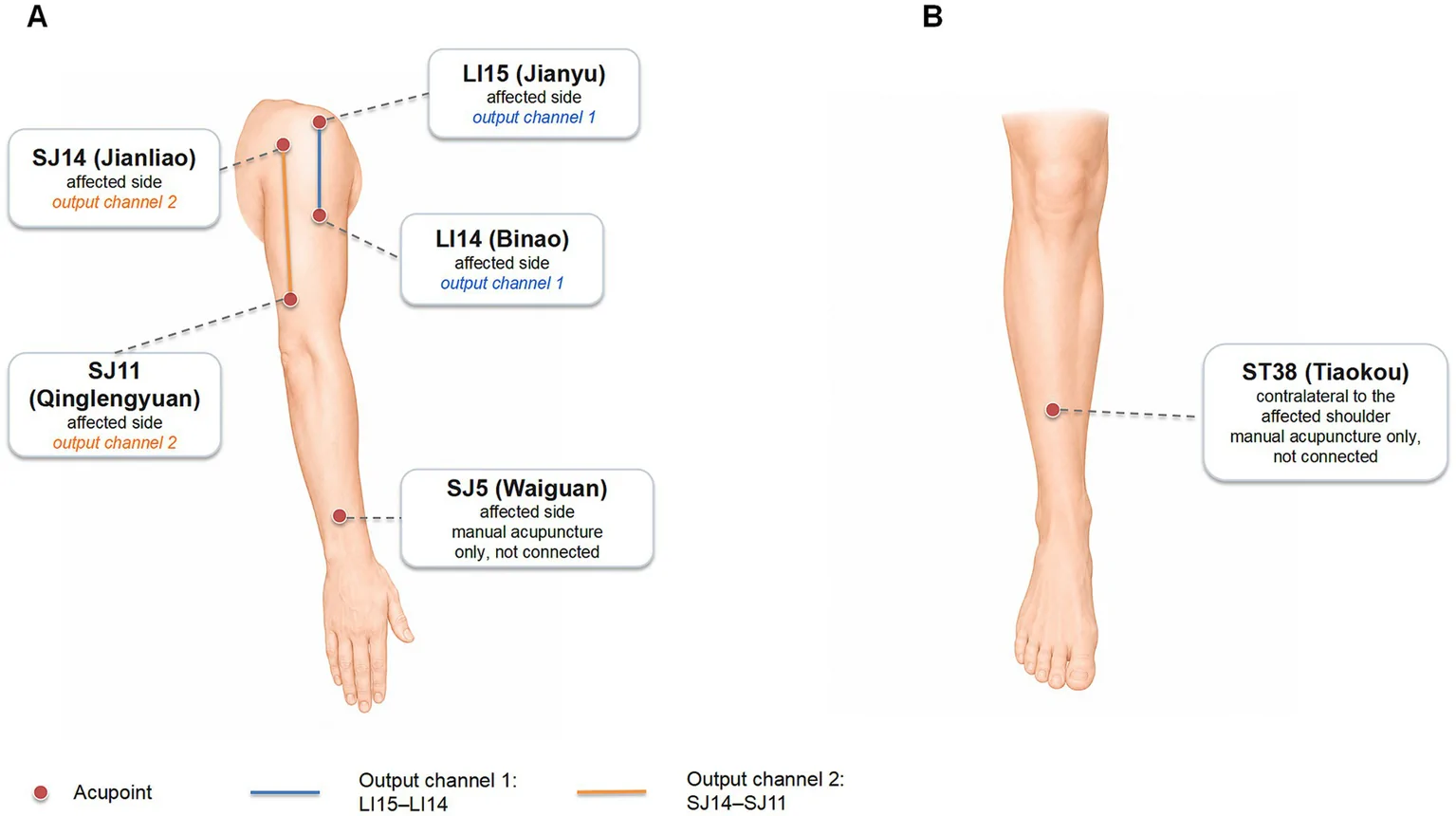
Acupoint distribution and electroacupuncture connection diagram. **(A)** Acupoints located on the affected shoulder and upper limb. Jianyu (LI15) and Binao (LI14) are connected to output channel 1 of the electroacupuncture device, while Jianliao (SJ14) and Qinglengyuan (SJ11) are connected to output channel 2. Waiguan (SJ5) is located on the affected side but is not connected to the electroacupuncture device and receives conventional needle retention only. **(B)** Tiaokou (ST38) is located on the lower limb contralateral to the affected shoulder and is not connected to the electroacupuncture device. Red dots indicate acupoints; blue and orange solid lines indicate electroacupuncture output channels 1 and 2, respectively; dashed lines indicate location reference or callout lines. LI, Large Intestine meridian; SJ, Sanjiao meridian; ST, Stomach meridian.

The acupuncture prescription was developed with reference to evidence-based acupuncture guidelines for shoulder periarthritis and commonly used clinical acupoint prescriptions. The acupoint prescription will be identical in both groups. The selected acupoints will include Jianyu (LI15), Binao (LI14), Jianliao (SJ14), Qinglengyuan (SJ11), and Waiguan (SJ5) on the affected side. Tiaokou (ST38) on the side contralateral to the affected shoulder will also be used. All acupoints will be located according to the WHO Standard Acupuncture Point Locations (25). The detailed acupoint locations and operating parameters are shown in Table 2. The acupoint distribution and EA connection method are shown in Figure 2.

**Table 2:** Acupoints and operating parameters for electroacupuncture treatment.

Acupoint	Meridian	Laterality	Location	Needling direction and depth	Manipulation	Electroacupuncture connection
LI15 (Jianyu)	Large Intestine meridian of hand-yangming	Affected side	On the shoulder, in the depression anterior and inferior to the acromion; when the arm is abducted, located in the depression between the anterior border of the acromion and the greater tubercle of the humerus.	Perpendicular insertion, approximately 0.8–1.5 cun.	Mainly reducing manipulation with twirling, aiming to elicit de qi sensations such as soreness, numbness, distension, heaviness, or local radiating sensation.	Output channel 1; connected with LI14.
LI14 (Binao)	Large Intestine meridian of hand-yangming	Affected side	On the lateral aspect of the upper arm, near the insertion of the deltoid muscle; located on the line connecting LI15 and LI11.	Perpendicular insertion, approximately 0.8–1.5 cun.	Mainly reducing manipulation with twirling, aiming to elicit de qi.	Output channel 1; connected with LI15.
SJ14 (Jianliao)	Sanjiao meridian of hand-shaoyang	Affected side	On the shoulder, in the depression posterior and inferior to the acromion when the arm is abducted.	Perpendicular insertion, approximately 0.8–1.2 cun.	Mainly reducing manipulation with twirling, aiming to elicit de qi.	Output channel 2; connected with SJ11.
SJ11 (Qinglengyuan)	Sanjiao meridian of hand-shaoyang	Affected side	On the posterior aspect of the upper arm, 2 cun proximal to the olecranon, on the line connecting the olecranon and SJ14.	Perpendicular insertion, approximately 0.5–1.0 cun.	Even reinforcing-reducing manipulation, aiming to elicit local soreness and distension.	Output channel 2; connected with SJ14.
SJ5 (Waiguan)	Sanjiao meridian of hand-shaoyang	Affected side	On the dorsal aspect of the forearm, 2 cun proximal to the dorsal wrist crease, between the radius and ulna.	Perpendicular insertion, approximately 0.5–1.0 cun.	Even reinforcing-reducing manipulation, aiming to elicit local soreness and distension.	Not connected to electroacupuncture; conventional needle retention only.
ST38 (Tiaokou)	Stomach meridian of foot-yangming	Contralateral to the affected shoulder	On the anterolateral aspect of the lower leg, 8 cun inferior to ST35 and one finger-breadth lateral to the anterior crest of the tibia.	Perpendicular insertion toward Chengshan (BL57), approximately 1.0–2.0 cun.	Mainly reducing manipulation with twirling, aiming to elicit de qi sensations such as soreness, numbness, distension, and heaviness.	Not connected to electroacupuncture; conventional needle retention only.

All acupoints will be located according to the WHO Standard Acupuncture Point Locations. Output channels 1 and 2 refer to two independent electrical output channels of the electroacupuncture device. In this study, output channel 1 will connect LI15 and LI14 on the affected side, and output channel 2 will connect SJ14 and SJ11 on the affected side. SJ5 on the affected side and ST38 contralateral to the affected shoulder will not be connected to the electroacupuncture device and will receive conventional needle retention only. Both groups will receive the same acupoint prescription, needling procedures, and electroacupuncture connection pattern; the only planned between-group difference will be the stimulation frequency (2 Hz vs. 100 Hz). BL, Bladder meridian; EA, electroacupuncture; LI, Large Intestine meridian; SJ, Sanjiao meridian; ST, Stomach meridian; TDP, specific electromagnetic spectrum lamp; WHO, World Health Organization; cun, a proportional body unit used in acupuncture point location; de qi, the characteristic needling sensation associated with acupuncture stimulation.

Needling procedures will be performed with reference to Acupuncture Therapeutics (24) and standard clinical practice. Disposable sterile filiform needles measuring 0.30 mm × 40 mm will be used. Jianyu (LI15) and Binao (LI14) will be inserted perpendicularly to a depth of approximately 0.8–1.5 cun. Jianliao (SJ14) will be inserted perpendicularly to a depth of approximately 0.8–1.2 cun. Tiaokou (ST38) will be inserted to a depth of approximately 1.0–2.0 cun, with the needle tip directed toward Chengshan (BL57). Qinglengyuan (SJ11) and Waiguan (SJ5) will be inserted perpendicularly to a depth of approximately 0.5–1.0 cun. The needling manipulation strategy was prespecified according to the therapeutic role of each acupoint in the prescription. Reducing manipulation with twirling will be mainly applied at the major local acupoints, including Jianyu (LI15), Binao (LI14), and Jianliao (SJ14), and at the distal shoulder-pain-related acupoint Tiaokou (ST38). Even reinforcing-reducing manipulation will be mainly applied at the auxiliary Shaoyang meridian acupoints Qinglengyuan (SJ11) and Waiguan (SJ5). The same manipulation strategy will be used in both groups to ensure intervention consistency. Appropriate needle sensations will include soreness, numbness, distension, heaviness, or local radiating sensation. Obvious sharp pain, burning pain, or intolerable discomfort will be avoided.

After needle insertion and the achievement of an appropriate needle sensation, an EA device will be connected for electrical stimulation. The same Huatuo SDZ-V electronic acupuncture treatment instrument (Suzhou Medical Appliance Factory Co., Ltd., Suzhou, China) will be used in both groups. Two independent output channels will be used. The first channel will connect Jianyu (LI15) and Binao (LI14) on the affected side. The second channel will connect Jianliao (SJ14) and Qinglengyuan (SJ11) on the affected side. Waiguan (SJ5) and Tiaokou (ST38) will not be connected to the EA device. These two acupoints will receive conventional needle retention only. Tiaokou (ST38) will be selected on the side contralateral to the affected shoulder. The needle tip will be directed toward Chengshan (BL57). The intended needle sensations will include soreness, numbness, distension, and heaviness.

Electrode clips and wires will be fixed in a standardized manner to avoid pulling, loosening, or poor contact. The SDZ-V device generates an asymmetric biphasic pulse waveform with a manufacturer-specified pulse width of 0.2 ms (tolerance, ±20%) and a frequency tolerance of ±20%. Continuous-wave stimulation will be used in both groups. The nominal frequency will be set at 2 Hz in the low-frequency EA group and 100 Hz in the high-frequency EA group.

Output intensity will be adjusted independently for each active channel using the device-defined scale ranging from 0 to 65. The device does not display the current delivered to the participant in milliamperes. Although the manufacturer specifies an output-current limit of ≤10 mA, this value represents the device limit and not the actual current delivered during an individual treatment session.

At the beginning of each session, the output level will be increased gradually from zero until a strong but tolerable stimulation sensation is achieved. Slight local muscle twitching or a participant-reported stimulation intensity of approximately 4–6 on a 0–10 scale will generally be considered appropriate. Sharp pain, burning pain, or intolerable discomfort will be avoided. If the stimulation sensation becomes markedly weaker or excessively strong during treatment, the practitioner may adjust the output level to maintain a relatively stable and tolerable sensation. Each EA session will last 30 min.

For each treatment session, the device-defined output level of each active channel will be recorded after the initial intensity has been stabilized and again at the end of the 30-min treatment period. Any adjustment made during the session, including the time, reason, and adjusted output level, will also be documented. The participant’s perceived stimulation intensity on the 0–10 scale will be recorded together with the device output levels.

The Massachusetts General Hospital Acupuncture Sensation Scale (MASS) (26) will be administered after each treatment session. The intensity of sensations such as soreness, numbness, distension, heaviness, and dull pain will be recorded on a 0–10 scale. This measure will not be used as a primary efficacy outcome but will be used for intervention process control, needle sensation recording, and adherence assessment.

To reduce nonspecific differences and maintain a consistent treatment background between groups, a TDP specific electromagnetic spectrum therapy device (Guoren L-I-5A; rated power, 250 W; nominal wavelength range, 2.5–20 μm) will be used to irradiate the affected shoulder during needle retention in both groups. Because both groups will receive the same TDP irradiation, this trial will mainly evaluate the relative efficacy of different EA frequencies under the same co-intervention background. The irradiation distance will be approximately 30–40 cm. The irradiation angle and distance will be kept as stable as possible during treatment. The intensity will be adjusted to produce a warm and comfortable sensation without burning. The duration of TDP irradiation will be the same as the needle retention time, namely 30 min. The practitioner will record the irradiation distance, participant tolerance, and any skin burning sensation, erythema, or other discomfort.

Treatment will be administered once daily. Five consecutive treatment days will constitute one treatment course. There will be a 2-day interval between courses. Two courses will be provided, for a total of 10 treatment sessions. All treatments should be completed according to the predefined schedule whenever possible. If the treatment time needs to be adjusted because of participant-related or objective reasons, an adjustment within a ±1-day window will be allowed. Any treatment delay, missed session, early termination, or other protocol deviation will be recorded in the case report form.

### 2.10 Concomitant care and rescue medication

During the study period, all participants should avoid any treatment other than the interventions specified in this protocol that may affect the assessment of shoulder pain, shoulder function, or shoulder ROM. Prohibited treatments include, but are not limited to, additional acupuncture, EA, moxibustion, cupping, tuina, acupotomy, miniscalpel-needle therapy, transcutaneous electrical stimulation, intra-articular shoulder injection, subacromial bursa injection, tendon sheath injection, nerve block, hydrodilatation, manipulation under anesthesia, arthroscopic release, and other interventional or surgical treatments targeting the affected shoulder. Except for the TDP lamp irradiation specified in this protocol, participants should not receive any other physical therapy targeting the affected shoulder. If any of these treatments is required for clinical reasons, the treatment type, timing, reason, and frequency will be recorded in detail. These cases will be reported as protocol deviations or concomitant treatments in the statistical analysis.

To reduce the influence of non-study interventions on efficacy assessment, participants will be advised not to initiate new shoulder rehabilitation exercises, intensive stretching, strength training, or other systematic shoulder treatments during the study period. Participants may maintain mild shoulder movement during daily activities, but should avoid excessive activity or strong stimulation that induces obvious pain. If basic home activity guidance is needed, standardized advice will be provided consistently to both groups. This advice will mainly include avoiding weight bearing with the affected shoulder, avoiding sudden traction, and avoiding shoulder movement beyond the pain-tolerable range. All relevant advice will be recorded and kept consistent between the two groups.

Participants will be allowed to continue regular medications unrelated to shoulder periarthritis if their underlying conditions are stable. These medications may include antihypertensive, antidiabetic, and lipid-lowering drugs. The type and dosage of these medications should remain as stable as possible. Any newly added medication, discontinued medication, or dose adjustment will be recorded, including the drug name, dose, frequency, start and end dates, and reason for use.

If participants experience intolerable shoulder pain during the study period, acetaminophen may be used as rescue analgesic medication. The recommended dose will be 0.5 g orally as needed, and the total daily dose should not exceed 2.0 g. Acetaminophen will be contraindicated in participants with severe hepatic dysfunction, allergy to acetaminophen, or other contraindications. Rescue medication use will be recorded in detail, including the date and time of administration, dose, frequency, reason for use, and perceived pain-relief response.

Participants will be advised to avoid rescue analgesic medication within 24 h before each scheduled outcome assessment whenever clinically feasible. However, necessary analgesic treatment will not be withheld because of study participation. If rescue medication is taken within the 24-h pre-assessment window, the scheduled outcome assessment will still be completed, and the timing and dose of medication will be documented. These outcome data will remain in the primary analysis and will be examined in prespecified sensitivity analyses.

If shoulder pain continues to worsen, marked functional deterioration occurs, or the study physician considers continued participation unsafe, routine clinical care or referral will be provided as clinically indicated. These situations will be recorded as protocol deviations, concomitant treatments, or AEs, as appropriate. Participants may withdraw from the study when necessary. The research team will make every effort to complete safety follow-up and record all relevant events that have occurred.

### 2.11 Outcome measurement

#### 2.11.1 Primary outcome

##### 2.11.1.1 Visual analog scale

The primary outcome will be overall shoulder pain intensity measured using a 10-cm VAS (27). The left endpoint of the scale represents “no pain,” and the right endpoint represents “the worst possible pain.” Research staff will provide the following standardized instruction: “Considering all periods of rest, movement, and nighttime during the past 24 h, please mark on the line the overall average intensity of pain in your affected shoulder.” The distance from the left endpoint to the participant’s mark will be measured to the nearest millimeter and expressed as a score ranging from 0 to 10, with a higher score indicating more severe pain. A sample of the VAS and its standardized administration instructions are provided in Supplementary Figure S1.

The overall VAS will be assessed at T0 (baseline/before randomization), T1 (end of treatment, 2 weeks after treatment initiation), T2 (short-term follow-up, 8 weeks after treatment initiation), and T3 (medium-term follow-up, 24 weeks after treatment initiation). The primary comparison will be the adjusted mean difference in the T1 overall VAS score between the two groups, with baseline overall VAS included as a covariate. Changes from baseline at T1, T2, and T3 will also be reported as supportive descriptive and follow-up efficacy outcomes.

To facilitate clinical interpretation, an individual reduction of ≥1.4 cm from baseline on the 10-cm overall VAS will be considered a minimal clinically important improvement (28). In addition, a post-treatment overall VAS score of ≤3.0 cm will be used as a supportive threshold for the patient acceptable symptomatic state (28). The proportions of participants achieving these thresholds at T1 will be evaluated as supportive exploratory outcomes.

#### 2.11.2 Secondary outcomes

##### 2.11.2.1 Component-specific pain intensity

In addition to the overall VAS assessment, resting pain, activity-related pain, and nocturnal pain will be evaluated separately using three 10-cm VAS ratings. For resting pain, participants will be asked to rate the average intensity of pain experienced while the affected shoulder was at rest during the preceding 24 h. For activity-related pain, participants will rate the average pain experienced during movement or use of the affected shoulder during the preceding 24 h. For nocturnal pain, participants will rate the average shoulder pain experienced during the previous night. Each scale will range from 0 (“no pain”) to 10 (“the worst possible pain”). These component-specific VAS ratings will be assessed at T0, T1, T2, and T3 and will be treated as exploratory secondary outcomes.

##### 2.11.2.2 Shoulder pain and disability index

The SPADI (29) will be used to assess shoulder pain and shoulder-related disability. The SPADI is a patient-reported outcome measure that includes two domains: pain and disability. Scores will be calculated according to the scoring instructions of the scale and converted to a 0–100 score. A higher score indicates more severe pain and disability. The SPADI will be assessed at T0, T1, T2, and T3.

##### 2.11.2.3 Constant–Murley score

The CMS (30) will be used to evaluate overall shoulder function. The CMS includes pain, activities of daily living, shoulder ROM, and strength. The total score ranges from 0 to 100, with a higher score indicating better shoulder function. The CMS will be assessed at T0, T1, T2, and T3 by trained and blinded outcome assessors.

##### 2.11.2.4 Shoulder range of motion

Shoulder ROM (31, 32) will be measured using a standard goniometer according to a standardized procedure. ROM will include AROM and PROM. The measured movements will include at least shoulder forward flexion, abduction, external rotation, internal rotation, and extension. All ROM measurements will be performed by trained and blinded outcome assessors according to a SOP. During measurement, participants will be placed in standardized positions. Each movement will be measured three consecutive times, and the mean value will be used as the final value for that movement. ROM will be assessed at T0, T1, T2, and T3.

#### 2.11.3 Safety outcomes

Safety outcomes will include AEs and SAEs occurring during treatment and follow-up. An AE is defined as any unfavorable or unexpected symptom, sign, disease, or laboratory or examination abnormality that occurs during the study period, regardless of whether it is causally related to the study intervention. An SAE is defined as an event that results in death, is life-threatening, requires hospitalization or prolongation of hospitalization, results in persistent or significant disability or functional impairment, or is considered medically important by the investigator.

Potential acupuncture-, EA-, or TDP-related AEs in this study include, but are not limited to, pain at the needling site, bleeding, subcutaneous hematoma, skin irritation, local erythema, burning discomfort, dizziness, fainting during acupuncture, infection, aggravation of shoulder pain, and other unexpected discomfort. Clinical research coordinators will record all AEs in detail, including onset time, duration, severity, management measures, outcome, and the investigator’s judgment regarding causality with the study intervention.

AE severity will be classified as mild, moderate, or severe. Mild AEs usually require no special management or only simple management. Moderate AEs may affect daily activities and require appropriate medical management. Severe AEs refer to events with marked symptoms that affect daily activities or require active medical management. All SAEs and important AEs judged by the investigator to be possibly related to the study intervention will be reported promptly according to the requirements of the ethics committee and the study institution. Serious adverse events will be reported to the principal investigator within 24 h after the research team becomes aware of the event and to the ethics committee and study institution as required.

Safety analysis will compare the incidence, type, severity, and relationship to the study intervention of AEs and SAEs between the two groups. Participants who experience AEs or SAEs related to the study intervention will receive appropriate medical management in a timely manner. Compensation or medical expense arrangements will be handled according to the ethics approval documents, the informed consent form, and relevant institutional regulations.

### 2.12 Data management and quality control

An electronic data capture system will be used to establish an electronic case report form (eCRF). The eCRF will record information on screening, enrollment, randomization, intervention delivery, outcome assessment, AEs, and follow-up. Each participant will be identified by a unique study identification number. Study data will be stored separately from personally identifiable information and will be accessible only to authorized research personnel. All data access, entry, modification, and export operations will be logged. These procedures will ensure data traceability, integrity, and confidentiality.

Before the study begins, the data manager will establish the eCRF data dictionary, variable naming rules, coding rules, range-checking rules, and logical-checking rules. These procedures will be based on the study protocol, outcome measures, and case report form content. Before data entry, all research personnel involved in data collection and entry will receive standardized training in variable definitions, scale scoring rules, missing-value coding, AE recording requirements, and data correction procedures. All original observation records, scales, imaging review results, treatment records, and follow-up records will be retained as source documents.

Research personnel will enter source document information into the eCRF within the specified time frame. The data manager will perform regular data checks, including completeness checks, range checks, logical consistency checks, and outlier checks. If missing, inconsistent, or abnormal data are identified, the data manager will issue a data query. The responsible research personnel will verify the data against the source documents and respond to the query. All data corrections will be documented, including the correction time, correction content, reason for correction, and the person who performed the correction. Original data must not be modified without documentation.

Outcome assessment will be performed by trained and blinded assessors. Assessors will not participate in participant treatment, have access to randomization results, or participate in statistical analysis. During the study period, the principal investigator will regularly organize quality control meetings to discuss participant recruitment, treatment adherence, data completeness, AEs, safety issues, and protocol deviations. Meeting records will be maintained. The research team will regularly review treatment records, follow-up completion, rescue medication records, and adverse event records to ensure that study implementation complies with the protocol.

Compliance with role separation and blinding procedures will be monitored throughout the trial. The principal investigator or designated quality-control personnel will periodically review the role-assignment and delegation log, treatment records, outcome-assessment records, electronic data-access permissions, and records of potential unblinding. Any unauthorized access, role overlap, communication of treatment allocation, or other event that may compromise blinding will be documented, including the date, personnel involved, reason, corrective action, and potential effect on study outcomes. Such events will be classified as protocol deviations and considered in sensitivity analyses and in the interpretation of the trial findings.

All study documents and electronic data will be stored at the designated study center or on a controlled server. Access control and regular backup measures will be implemented. After database cleaning is completed and confirmed by the principal investigator, data manager, and statistician, the database will be locked. After database locking, the database must not be modified unless major data issues are identified that may affect interpretation of the study results. If modification is necessary, it must be approved by the principal investigator, and the reason, content, and time of modification must be fully documented. Study-related records will be retained according to the requirements of the ethics committee and the study institution.

### 2.13 Statistical analysis

Statistical analysis will be performed after database locking according to a prespecified statistical analysis plan. SPSS version 28.0 will be used for statistical analysis. R software may be used for supplementary analyses when necessary. All statistical tests will be two-sided. A *p* value <0.05 will be considered statistically significant. Effect sizes, 95% confidence intervals, and *p* values will be reported for the primary efficacy results. Except for the primary outcome, analyses of secondary outcomes and follow-up outcomes will be mainly exploratory.

The following analysis sets will be defined in this trial:

- Full analysis set (FAS): The FAS will include all randomized participants who have at least one post-baseline assessment of the primary outcome. Participants will be analyzed according to their randomized groups. The primary efficacy analysis will be based on the FAS and will follow a modified intention-to-treat principle;
- Per-protocol set (PPS): The PPS will include participants who complete the main treatment course, have primary outcome data, have no major protocol deviations, and show protocol-compliant adherence. The PPS will be used for sensitivity analysis of the primary outcome;
- Safety set (SS): The SS will include all participants who receive at least one study intervention and have safety records. Safety analysis will be based on the SS.

Baseline characteristics will be summarized using descriptive statistics. Continuous variables will be presented as the mean ± standard deviation or as the median with interquartile range, depending on data distribution. Categorical variables will be presented as counts and percentages. Baseline balance between the two groups will be described; however, baseline significance testing will not be used as the main criterion for judging the success of randomization.

The primary efficacy endpoint will be the T1 overall VAS score. The primary analysis will use an analysis of covariance model. The T1 VAS score will be used as the dependent variable, group will be included as a fixed factor, and baseline VAS score will be included as a covariate. The adjusted mean difference in T1 VAS score between the two groups will be reported with the 95% confidence interval and *p* value. Descriptive results for the change from baseline to T1 will also be reported. At T1, the proportions of participants achieving a reduction of ≥1.4 cm from baseline in the overall VAS and an overall VAS score of ≤3.0 cm will be summarized by treatment group. Between-group differences will be evaluated using the chi-square test or Fisher’s exact test, as appropriate. Risk ratios and risk differences will be reported with 95% confidence intervals. These responder analyses will be considered supportive and exploratory.

To evaluate treatment trends over time, linear mixed-effects models will be used for continuous repeated-measure outcomes, including the overall VAS, component-specific VAS ratings, SPADI, CMS, and ROM. The models will include group, time, group-by-time interaction, and the corresponding baseline value. Participant will be included as a random effect. Time will be treated as a categorical variable. An unstructured covariance structure will be preferred. If the model does not converge, an appropriate covariance structure will be selected according to model fit indices. The group-by-time interaction will be used to evaluate whether treatment trends differ between the two groups across time points. The between-group difference in the primary outcome at T1 will be the main focus. Between-group differences in changes from baseline at T2 and T3 will also be reported for each outcome.

The component-specific VAS ratings for resting pain, activity-related pain, and nocturnal pain will be analyzed as exploratory secondary outcomes. Between-group differences at T1 will be estimated using analysis of covariance with adjustment for the corresponding baseline pain score. Changes over time will be explored using linear mixed-effects models including group, time, group-by-time interaction, and the corresponding baseline value. Effect estimates and 95% confidence intervals will be reported, and these analyses will be interpreted as exploratory.

Secondary outcomes, including SPADI, CMS, and ROM, will be analyzed using appropriate statistical methods according to variable type and data distribution. Continuous outcomes will be analyzed preferentially using analysis of covariance or linear mixed-effects models. If the data are clearly non-normally distributed, nonparametric tests or appropriate data transformation may be used. Categorical variables will be analyzed using the chi-square test or Fisher’s exact test. Ordinal data will be analyzed using rank-sum tests. Adverse events and SAEs will be described by number, incidence, type, severity, and relationship to the study intervention. Differences in incidence between the two groups will also be compared.

The device-defined output levels recorded after initial stabilization and at the end of each treatment session, the frequency of within-session adjustments, and participant-reported stimulation intensity will be summarized descriptively by treatment group and session. Exploratory analyses may examine associations between recorded stimulation intensity and changes in pain outcomes, with adjustment for treatment group and baseline pain where appropriate. Because stimulation intensity is individually titrated and is not a randomized exposure, these analyses will not be interpreted as evidence of a causal intensity effect.

For missing data, the reason, time point, and type of missingness will first be recorded. In the longitudinal repeated-measures analyses, linear mixed-effects models will use available repeated-measure data under reasonable assumptions about the missing data mechanism. If the proportion of missing data for the primary outcome is high, multiple imputation will be used for sensitivity analysis. The influence of different missing data handling methods on the study conclusions will be compared. Simple last observation carried forward will not be used as the primary method for handling missing data.

Sensitivity analyses will be performed to assess the robustness of the primary findings. These analyses will include an analysis based on the PPS, an analysis excluding participants with major protocol deviations, and analyses addressing rescue analgesic use. The primary analysis will include all available T1 VAS measurements regardless of rescue medication use and will not routinely adjust for post-randomization rescue medication use. The primary analysis will then be repeated after excluding assessments preceded by rescue medication use within 24 h. A further sensitivity analysis will include pre-assessment rescue medication use and the corresponding dose as additional covariates in the analysis of covariance model. The number and proportion of participants using rescue medication, cumulative acetaminophen dose, and number of days of use will be summarized by treatment group. If participants receive prohibited concomitant treatments or experience events that may substantially affect efficacy assessment during the trial, these situations will be documented as protocol deviations and considered in sensitivity analyses.

### 2.14 Patient and public involvement, trial oversight, monitoring, and open science

No patient or public representatives were involved in the initial design of this trial protocol. Participant feedback collected during recruitment, treatment, and follow-up will be considered when refining future multicenter studies or optimizing the acceptability of the intervention procedures.

Changchun University of Chinese Medicine is listed as the applicant institution, and the Third Clinical Hospital Affiliated to Changchun University of Chinese Medicine will serve as the clinical study site and coordinating center for this single-center trial. Trial-related administrative matters will be coordinated by the study site and the principal investigator. The principal investigator, Yi Shang, will oversee the overall conduct of the trial. The coordinating center will be responsible for participant recruitment, intervention delivery, outcome assessment, safety management, data collection, and document storage. The data management team and independent statistician will be responsible for eCRF development, data checking, database locking, and statistical analysis. The funder will have no role in study design, trial conduct, data collection, data management, statistical analysis, interpretation of results, manuscript preparation, or the decision to submit the manuscript for publication.

A formal independent data monitoring committee will not be established because this is a single-center, low-risk, non-drug trial with a short treatment period. Safety and trial conduct will be reviewed by the principal investigator and the study team at regular quality-control meetings. No interim efficacy analysis is planned. The study may be temporarily suspended or terminated early in the event of unexpected safety concerns, serious protocol noncompliance, severe recruitment difficulty, or a decision by the ethics committee or study institution.

Important protocol amendments will be submitted to the ethics committee for approval before implementation and updated in the trial registry when applicable. Relevant amendments will be communicated to investigators, outcome assessors, data managers, and other trial personnel. If an amendment affects participant rights, safety, or study procedures, the informed consent form and other participant-facing materials will be revised accordingly.

The full trial protocol is reported in this manuscript. The detailed statistical analysis plan will be finalized before database locking and will be available from the corresponding authors on reasonable request. After publication of the trial results, de-identified participant-level data, the data dictionary, and relevant statistical code underlying the published findings may be made available from the corresponding authors on reasonable request, subject to ethics and institutional approval.

## 3 Discussion

This study protocol describes a single-center, participant- and outcome assessor-blinded, parallel-group RCT. The trial will compare the efficacy and safety of 2 Hz and 100 Hz EA for pain-dominant shoulder periarthritis. Previous studies have mainly examined whether acupuncture or EA is effective for shoulder pain or frozen shoulder. This trial focuses on a more specific question: how EA frequency should be selected in this clinical stage. Under the same acupuncture prescription, treatment procedure, and co-intervention setting, the trial will compare the potential effects of two stimulation frequencies on pain relief, shoulder function, and range-of-motion recovery.

The trial will enroll patients with pain-dominant shoulder periarthritis and the Hand Shaoyang meridian pattern. This restriction is intended to improve clinical homogeneity. Shoulder periarthritis has a staged clinical course, and the main therapeutic problem may differ across stages. In the pain-dominant stage, pain, nocturnal pain, and worsening movement limitation are the major clinical concerns. This stage is therefore suitable for evaluating early analgesia and functional preservation. The additional use of TCM meridian differentiation may further reduce heterogeneity in acupoint selection and clinical presentation. It also reflects the diagnostic and therapeutic logic commonly used in acupuncture practice.

### 3.1 Acupoint selection rationale

The acupoint prescription in this trial follows the principles of local acupoint selection, meridian-based acupoint selection, and distal acupoint selection. Jianyu (LI15), Binao (LI14), and Jianliao (SJ14) are located around the shoulder joint and proximal upper arm. These acupoints are close to the main sites of pain and movement limitation and are consistent with the clinical principle of local acupoint selection for shoulder periarthritis. Jianyu (LI15) and Jianliao (SJ14) have also been frequently used in previous acupuncture studies on frozen shoulder (13).

Qinglengyuan (SJ11) and Waiguan (SJ5) are associated with the Hand Shaoyang meridian. Their use is consistent with the inclusion of participants with the Hand Shaoyang meridian pattern. Tiaokou (ST38) is a commonly used distal acupoint for shoulder pain and restricted shoulder movement. It reflects the principle of distal acupoint selection and helps form a local-distal acupoint combination with the shoulder acupoints (33). Therefore, this trial uses a fixed acupoint prescription that is consistent with TCM meridian differentiation and common clinical acupoint selection. This approach may reduce heterogeneity caused by individualized acupoint selection and improve the standardization and reproducibility of the intervention.

### 3.2 Analgesic rationale for comparing 2 Hz and 100 Hz EA

Electroacupuncture frequency is one of the most easily quantified and standardized parameters in EA treatment. It is also an important variable that may influence analgesic effects. Previous studies have suggested that different EA frequencies may act through different endogenous opioid peptides, opioid receptors, and descending pain-modulatory mechanisms. Low-frequency EA is more closely related to *μ*- and *δ*-opioid receptor-mediated mechanisms, whereas high-frequency EA may involve *κ*-opioid receptor-related pathways (16–20).

In clinical research, direct comparisons between single-frequency 2 Hz and 100 Hz EA remain limited. Existing evidence mainly comes from studies on postoperative pain (34), chronic low back pain or lumbar osteoarthritis (35, 36), and chemotherapy-induced peripheral neuropathy (37). Preclinical studies using tail-flick, ankle sprain, post-incision pain, diabetic neuropathic pain, inflammatory pain, spared nerve injury, and nerve cuffing models also suggest that 2 Hz and 100 Hz EA may show model-dependent analgesic profiles (17, 20, 38–44). Relevant evidence from clinical trials and animal studies is summarized in Table 3.

**Table 3:** Clinical and preclinical evidence related to the analgesic effects of 2 Hz and 100 Hz electroacupuncture.

Reference	Disease type/pain model	Frequency setting	Main findings
Lin et al.^a^ (34)	Postoperative pain after lower abdominal surgery	2 Hz, 100 Hz, sham EA, and control	Both 2 Hz and 100 Hz EA reduced postoperative analgesic requirements. The 100 Hz regimen appeared to show greater effects on some analgesic outcomes.
Trinh et al.^a^ (35)	Chronic low back pain related to lumbar osteoarthritis	100 Hz vs. 2 Hz	The 100 Hz group showed a greater reduction in pain score and a higher pain-relief proportion than the 2 Hz group.
Torres et al.^a^ (36)	Chronic nonspecific low back pain in older adults	100 Hz, 2 Hz, alternating 2/100 Hz, manual acupuncture, and placebo control	No EA frequency showed clear superiority. Pain intensity decreased in all groups, and EA was not superior to manual acupuncture or placebo.
Lu et al.^a^ (37)	Paclitaxel-induced peripheral neuropathy	2 Hz, 100 Hz, alternating 2/100 Hz, and medication control	Different EA frequency regimens improved some symptoms. The 2 Hz and alternating 2/100 Hz regimens showed potential advantages in some sensory or pain-related outcomes.
Chen and Han^b^ (17)	Acute nociceptive pain in rats (tail-flick test)	2 Hz, 100 Hz, and 2–15 Hz	Analgesia induced by 2 Hz and 100 Hz EA may be mediated by different opioid receptor mechanisms.
Han et al.^b^ (38)	Acute nociceptive pain in rats (tail-flick test)	2 Hz vs. 100 Hz	Endomorphin-1 mediated 2 Hz, but not 100 Hz, EA-induced analgesia.
Hahm^b^ (39)	Rat ankle sprain model	2 Hz vs. 100 Hz	Both 2 Hz and 100 Hz stimulation produced analgesic effects. The 2 Hz regimen showed a more marked effect on edema.
Fais et al.^b^ (40)	Rat post-incision pain model	2 Hz vs. 100 Hz	Both 2 Hz and 100 Hz EA produced antihyperalgesic effects. The 2 Hz effect lasted longer, whereas amitriptyline potentiated the 100 Hz effect more strongly.
Silva et al.^b^ (41)	Rat post-incision pain model	2 Hz and 100 Hz	Preoperative 100 Hz EA showed a preemptive effect against post-incision pain, whereas postoperative 2 Hz EA appeared more effective.
He et al.^b^ (42)	Type 2 diabetic neuropathic pain in rats	2 Hz vs. 100 Hz	Both 2 Hz and 100 Hz EA alleviated diabetic neuropathic pain, but 2 Hz produced stronger analgesic effects.
Fang et al.^b^ (43)	CFA-induced inflammatory pain in rats	2 Hz, 100 Hz, and 2/100 Hz	Different EA frequencies produced analgesic effects. The 100 Hz effect was stronger than the 2 Hz effect and was associated with TRPV1/P2X3 regulation.
Du et al.^b^ (44)	SNI-induced neuropathic pain in rats	2 Hz vs. 100 Hz	Both 2 Hz and 100 Hz EA improved neuropathic pain. The 2 Hz regimen showed stronger modulation of TRPV1/P2X3 expression.

Superscript a indicates a clinical trial, and superscript b indicates an animal study. CFA, complete Freund’s adjuvant; EA, electroacupuncture; P2X3, purinergic receptor P2X3; SNI, spared nerve injury; TRPV1, transient receptor potential vanilloid 1.

These findings indicate that both 2 Hz and 100 Hz EA have a basis in analgesic research. However, the relative advantages of different frequencies may vary according to pain type, disease stage, stimulation timing, and outcome measures. In pain-dominant shoulder periarthritis, direct clinical trial evidence comparing 2 Hz and 100 Hz EA remains lacking. This head-to-head trial will address this specific evidence gap and may help clarify frequency selection for EA in this disease stage.

### 3.3 Intervention standardization and outcome selection

This trial will keep all intervention factors as consistent as possible between the two groups, except for EA frequency. These factors include acupoints, needle specifications, insertion methods, de qi requirements, connected acupoints, waveform, needle retention time, treatment frequency, TDP irradiation, and treatment setting. This design will help isolate EA frequency as the key variable and reduce confounding from other treatment factors.

Both groups will receive the same TDP irradiation. Therefore, this trial will evaluate the relative efficacy of 2 Hz and 100 Hz EA under the same co-intervention setting. It will not evaluate the independent effect of TDP or the absolute efficacy of EA compared with no treatment, usual care, or sham acupuncture.

The VAS is selected as the primary outcome because pain is the most direct and core clinical manifestation of pain-dominant shoulder periarthritis, and it provides a simple validated measure of pain intensity (27). SPADI, CMS, and ROM will be used as secondary outcomes. These measures evaluate patient-reported pain and disability, overall shoulder function, and AROM and PROM, respectively (29–32). This outcome set can capture subjective pain experience, functional limitation, and recovery of shoulder mobility, thereby providing a more comprehensive evaluation than a single pain outcome.

This trial will use several follow-up time points. T1 corresponds to the end of treatment and will mainly assess early analgesic effects after the treatment course. T2 and T3 will be used to observe changes in pain, function, and ROM during short- and medium-term follow-up. Shoulder periarthritis has a staged clinical course, and pain relief, functional improvement, and ROM recovery may not occur at the same pace. Repeated assessments at multiple time points may help characterize the time course of treatment effects for different EA frequencies and provide evidence for evaluating the durability of treatment effects.

### 3.4 Strengths and limitations

This trial has several strengths. First, it focuses on a clinically specific population, namely patients with pain-dominant shoulder periarthritis. Second, it directly compares two representative EA frequencies under otherwise standardized intervention conditions. Third, participant blinding, outcome assessor blinding, allocation concealment, and prespecified statistical analysis will be used to reduce bias. Fourth, the use of VAS, SPADI, CMS, and ROM will allow multidimensional evaluation of pain, function, and movement recovery.

Several limitations should also be acknowledged. First, this is a single-center trial, which may limit the generalizability of the findings. Second, treating acupuncturists cannot be blinded because they must set the EA frequency. This may introduce performance-related bias, although treating acupuncturists will not participate in outcome assessment or data analysis. Third, although participants will not be informed of their treatment allocation, the temporal sensations produced by 2 Hz and 100 Hz stimulation may differ, and complete participant blinding cannot be guaranteed. The success of participant blinding will therefore be assessed at T1 and considered when interpreting the findings. Fourth, both groups will receive the same TDP irradiation. The trial can therefore evaluate the relative effects of two EA frequencies, but not the independent effect of EA alone. Fifth, a fixed acupoint prescription improves standardization but may not fully reflect individualized acupuncture practice. Finally, the sample size is designed for the primary pain outcome and may not be sufficient to detect rare AEs or small differences in secondary outcomes.

In summary, this trial will compare 2 Hz and 100 Hz EA for pain-dominant shoulder periarthritis using a standardized randomized controlled design. The study will focus on early pain relief, shoulder function, range-of-motion recovery, and safety. The findings may provide evidence for EA frequency selection and parameter standardization in this clinical stage. They may also inform future multicenter trials and the optimization of acupuncture treatment protocols for shoulder periarthritis.

## 4 Conclusion

This RCT is designed to compare the efficacy and safety of 2 Hz and 100 Hz EA in patients with pain-dominant shoulder periarthritis. The findings may provide clinical evidence for EA frequency selection in this disease stage. They may also inform parameter optimization and the standardized application of EA in future clinical practice and research.
