# Supplementary material for: Efficacy and safety of 2 Hz versus 100 Hz electroacupuncture for pain-dominant shoulder periarthritis: a study protocol for a single-center randomized controlled trial
Source: Front Med (Lausanne). 2026 Aug 27;13:1924469. doi: 10.3389/fmed.2026.1924469 (PMC13558102; doi:10.3389/fmed.2026.1924469)
Supplement: Supplementary file 1 [file Table_1.docx]

**SPIRIT 2025 checklist of items to address in a randomized trial protocol***

| **Section / Topic** | **No** | **SPIRIT 2025 checklist item description** | **Location in manuscript (section/subsection)** |
| --- | --- | --- | --- |
| **Administrative information** | | |  |
| Title and structured summary | 1a | Title stating the trial design, population, and interventions, with identification as a protocol | Title page |
|  | 1b | Structured summary of trial design and methods, including items from the World Health Organization Trial Registration Data Set | Abstract |
| Protocol version | 2 | Version date and identifier | Title page (Protocol version) |
| Roles and responsibilities | 3a | Names, affiliations, and roles of protocol contributors | Title page; Author contributions |
|  | 3b | Name and contact information for the trial sponsor | Title page (Correspondence); Patient and public involvement, trial oversight, monitoring, and open science |
|  | 3c | Role of trial sponsor and funders in design, conduct, analysis, and reporting of trial; including any authority over these activities | Patient and public involvement, trial oversight, monitoring, and open science; Funding |
|  | 3d | Composition, roles, and responsibilities of the coordinating site, steering committee, endpoint adjudication committee, data management team, and other individuals or groups overseeing the trial, if applicable | Data management and quality control; Patient and public involvement, trial oversight, monitoring, and open science |
| **Open science** | | |  |
| Trial registration | 4 | Name of trial registry, identifying number (with URL), and date of registration. If not yet registered, name of intended registry | Abstract (Clinical Trial Registration); Study design |
| Protocol and statistical analysis plan | 5 | Where the trial protocol and statistical analysis plan can be accessed | Patient and public involvement, trial oversight, monitoring, and open science |
| Data sharing | 6 | Where and how the individual de-identified participant data (including data dictionary), statistical code, and any other materials will be accessible | Patient and public involvement, trial oversight, monitoring, and open science |
| Funding and conflicts of interest | 7a | Sources of funding and other support (e.g., supply of drugs) | Funding |
|  | 7b | Financial and other conflicts of interest for principal investigators and steering committee members | Conflict of interest |
| Dissemination policy | 8 | Plans to communicate trial results to participants, healthcare professionals, the public, and other relevant groups (e.g., reporting in trial registry, plain language summary, publication) | Ethics and Dissemination |
| **Introduction** | | |  |
| Background and rationale | 9a | Scientific background and rationale, including summary of relevant studies (published and unpublished) examining benefits and harms for each intervention | Introduction; Analgesic rationale for comparing 2 Hz and 100 Hz EA; Table 3 |
|  | 9b | Explanation for choice of comparator | Introduction; Analgesic rationale for comparing 2 Hz and 100 Hz EA; Intervention standardization and outcome selection |
| Objectives | 10 | Specific objectives related to benefits and harms | Abstract; Introduction; Study design |
| **Methods: Patient and public involvement, trial design** | | |  |
| Patient and public involvement | 11 | Details of, or plans for, patient or public involvement in the design, conduct, and reporting of the trial | Patient and public involvement, trial oversight, monitoring, and open science |
| Trial design | 12 | Description of trial design including type of trial (e.g., parallel group, crossover), allocation ratio, and framework (e.g., superiority, equivalence, non-inferiority, exploratory) | Study design |
| **Methods: Participants, interventions, and outcomes** | | |  |
| Trial setting | 13 | Settings (e.g., community, hospital) and locations (e.g., countries, sites) where the trial will be conducted | Study design; Recruitment process |
| Eligibility criteria | 14a | Eligibility criteria for participants | Diagnostic criteria; Western medical diagnostic criteria; TCM meridian differentiation criteria; Inclusion criteria; Exclusion criteria |
|  | 14b | If applicable, eligibility criteria for sites and for individuals who will deliver the interventions (e.g., surgeons, physiotherapists) | Intervention; Patient and public involvement, trial oversight, monitoring, and open science |
| Intervention and comparator | 15a | Intervention and comparator with sufficient details to allow replication including how, when, and by whom they will be administered. If relevant, where additional materials describing the intervention and comparator (e.g., intervention manual) can be accessed | Intervention; Concomitant care and rescue medication; Table 1; Table 2; Figure 2 |
|  | 15b | Criteria for discontinuing or modifying allocated intervention/comparator for a trial participant (e.g., drug dose change in response to harms, participant request, or improving/worsening disease) | Recruitment process; Intervention; Concomitant care and rescue medication |
|  | 15c | Strategies to improve adherence to intervention/comparator protocols, if applicable, and any procedures for monitoring adherence (e.g., drug tablet return, sessions attended) | Intervention; Data management and quality control; Table 1 |
|  | 15d | Concomitant care that is permitted or prohibited during the trial | Concomitant care and rescue medication |
| Outcomes | 16 | Primary and secondary outcomes, including the specific measurement variable (e.g., systolic blood pressure), analysis metric (e.g., change from baseline, final value, time to event), method of aggregation (e.g., median, proportion), and time point for each outcome | Study design; Outcome measurement; Statistical analysis; Table 1 |
| Harms | 17 | How harms are defined and will be assessed (e.g., systematically, non-systematically) | Safety outcomes; Table 1 |
| Participant timeline | 18 | Time schedule of enrollment, interventions (including any run-ins and washouts), assessments, and visits for participants. A schematic diagram is highly recommended (see Figure) | Study design; Recruitment process; Intervention; Table 1; Figure 1 |
| Sample size | 19 | How sample size was determined, including all assumptions supporting the sample size calculation | Sample size calculation |
| Recruitment | 20 | Strategies for achieving adequate participant enrollment to reach target sample size | Recruitment process |
| **Methods: Assignment of interventions** | | |  |
| Randomization: |  |  |  |
| Sequence generation | 21a | Who will generate the random allocation sequence and the method used | Randomization and allocation concealment |
|  | 21b | Type of randomization (simple or restricted) and details of any factors for stratification. To reduce predictability of a random sequence, other details of any planned restriction (e.g., blocking) should be provided in a separate document that is unavailable to those who enroll participants or assign interventions | Randomization and allocation concealment |
| Allocation concealment  mechanism | 22 | Mechanism used to implement the random allocation sequence (e.g., central computer/telephone; sequentially numbered, opaque, sealed containers), describing any steps to conceal the sequence until interventions are assigned | Randomization and allocation concealment |
| Implementation | 23 | Whether the personnel who will enroll and those who will assign participants to the interventions will have access to the random allocation sequence | Randomization and allocation concealment; Blinding |
| Blinding | 24a | Who will be blinded after assignment to interventions (e.g., participants, care providers, outcome assessors, data analysts) | Blinding |
|  | 24b | If blinded, how blinding will be achieved and description of the similarity of interventions | Blinding; Intervention |
|  | 24c | If blinded, circumstances under which unblinding is permissible, and procedure for revealing a participant’s allocated intervention during the trial | Blinding |
| **Methods: Data collection, management, and analysis** | | |  |
| Data collection methods | 25a | Plans for assessment and collection of trial data, including any related processes to promote data quality (e.g., duplicate measurements, training of assessors) and a description of trial instruments (e.g., questionnaires, laboratory tests) along with their reliability and validity, if known. Reference to where data collection forms can be accessed, if not in the protocol | Intervention; Outcome measurement; Data management and quality control; Table 1; Supplementary Figure S1 |
|  | 25b | Plans to promote participant retention and complete follow-up, including list of any outcome data to be collected for participants who discontinue or deviate from intervention protocols | Recruitment process; Intervention; Concomitant care and rescue medication; Data management and quality control |
| Data management | 26 | Plans for data entry, coding, security, and storage, including any related processes to promote data quality (e.g., double data entry; range checks for data values). Reference to where details of data management procedures can be accessed, if not in the protocol | Data management and quality control |
| Statistical methods | 27a | Statistical methods used to compare groups for primary and secondary outcomes, including harms | Statistical analysis |
|  | 27b | Definition of who will be included in each analysis (e.g., all randomized participants), and in which group | Statistical analysis |
|  | 27c | How missing data will be handled in the analysis | Statistical analysis |
|  | 27d | Methods for any additional analyses (e.g., subgroup and sensitivity analyses) | Statistical analysis |
| **Methods: Monitoring** | | |  |
| Data monitoring committee | 28a | Composition of data monitoring committee (DMC); summary of its role and reporting structure; statement of whether it is independent from the sponsor and funder; conflicts of interest and reference to where further details about its charter can be found, if not in the protocol. Alternatively, an explanation of why a DMC is not needed | Patient and public involvement, trial oversight, monitoring, and open science |
|  | 28b | Explanation of any interim analyses and stopping guidelines, including who will have access to these interim results and make the final decision to terminate the trial | Patient and public involvement, trial oversight, monitoring, and open science |
| Trial monitoring | 29 | Frequency and procedures for monitoring trial conduct. If there is no monitoring, give explanation | Data management and quality control; Patient and public involvement, trial oversight, monitoring, and open science |
| **Ethics** | | |  |
| Research ethics approval | 30 | Plans for seeking research ethics committee/institutional review board approval | Study design; Ethics and Dissemination |
| Protocol amendments | 31 | Plans for communicating important protocol modifications to relevant parties | Patient and public involvement, trial oversight, monitoring, and open science |
| Consent or assent | 32a | Who will obtain informed consent or assent from potential trial participants or authorized proxies, and how | Study design; Recruitment process; Ethics and Dissemination |
|  | 32b | Additional consent provisions for collection and use of participant data and biological specimens in ancillary studies, if applicable | Not applicable |
| Confidentiality | 33 | How personal information about potential and enrolled participants will be collected, shared, and maintained in order to protect confidentiality before, during, and after the trial | Study design; Recruitment process; Data management and quality control |
| Ancillary and post-trial care | 34 | Provisions, if any, for ancillary and post-trial care, and for compensation to those who suffer harm from trial participation | Concomitant care and rescue medication; Safety outcomes |

*We strongly recommend reading this checklist in conjunction with the SPIRIT 2025 Explanation and Elaboration and the SPIRIT 2025 Expanded Checklist for important clarifications on all the items. We also recommend reading relevant SPIRIT extensions. See [www.consort-spirit.org](http://www.consort-spirit.org)

Citation: Chan A-W, Boutron I, Hopewell S, Moher D, Schulz KF, et al. SPIRIT 2025 statement: updated guideline for protocols of randomised trials. BMJ 2025;389:e081477. <https://dx.doi.org/10.1136/bmj-2024-081477>

© 2025 Chan A-W et al. This is an Open Access article distributed under the terms of the Creative Commons Attribution License (<https://creativecommons.org/licenses/by/4.0/>), which permits unrestricted use, distribution, and reproduction in any medium, provided the original work is properly cited.
